# Single crystal functional oxides on silicon

**DOI:** 10.1038/ncomms10547

**Published:** 2016-02-08

**Authors:** Saidur Rahman Bakaul, Claudy Rayan Serrao, Michelle Lee, Chun Wing Yeung, Asis Sarker, Shang-Lin Hsu, Ajay Kumar Yadav, Liv Dedon, Long You, Asif Islam Khan, James David Clarkson, Chenming Hu, Ramamoorthy Ramesh, Sayeef Salahuddin

**Affiliations:** 1 Department of Electrical Engineering and Computer Sciences, University of California, Berkeley, California, USA; 2 Department of Material Science and Engineering, University of California, Berkeley, California, USA; 3 Department of Physics, University of California, Berkeley, California, USA; 4 Material Science Division, Lawrence Berkeley National Laboratory, Berkeley, California, USA

## Abstract

Single-crystalline thin films of complex oxides show a rich variety of functional properties such as ferroelectricity, piezoelectricity, ferro and antiferromagnetism and so on that have the potential for completely new electronic applications. Direct synthesis of such oxides on silicon remains challenging because of the fundamental crystal chemistry and mechanical incompatibility of dissimilar interfaces. Here we report integration of thin (down to one unit cell) single crystalline, complex oxide films onto silicon substrates, by epitaxial transfer at room temperature. In a field-effect transistor using a transferred lead zirconate titanate layer as the gate insulator, we demonstrate direct reversible control of the semiconductor channel charge with polarization state. These results represent the realization of long pursued but yet to be demonstrated single-crystal functional oxides on-demand on silicon.

## Abstract (web summary)

Synthesis of single-crystal complex-oxide films directly on silicon is difficult due to differing interfacial chemistry. Here, the authors demonstrate room-temperature integration of single-crystal lead zirconate titanate on to silicon to act as a gate insulator in a field-effect transistor.

## Graphical abstract

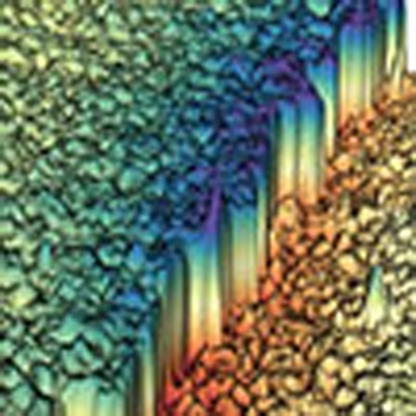

A significant number of single-crystalline complex oxides show ferroic order and a variety of correlated phenomena^1^,^2^. Consequently, extensive research effort is currently ongoing in the investigation of these materials both for fundamental science and potential applications. For many of the novel functionalities, it is important to retain the single-crystal nature of these oxides when they are finally interfaced with Si electronics. In addition, it has been long postulated that integration of single-crystal functional oxides with silicon could resolve some of the most critical problems in existing applications such as the memory retention time in ferroelectric random access memory^3^. As a consequence, there is currently a significant effort to integrate functional complex oxides on silicon^4^,^5^,^6^,^7^,^8^,^9^,^10^,^11^,^12^,^13^,^14^,^15^,^16^,^17^. However, owing to large difference in interfacial chemistry and the typically high temperatures and oxidizing environments needed for the growth of such oxides, direct epitaxial synthesis on Si continues to pose a significant synthesis challenge^6^,^7^,^8^,^9^,^10^. Such integration is mostly achieved by growing an appropriate buffer layer^9^,^11^,^12^,^13^,^14^,^15^,^16^, which then acts as a template for synthesis of subsequent layers either by epitaxy or other techniques. Synthesis of a ferroelectric without a buffer layer has also been demonstrated^17^. However, a common problem in all these methods comes from the electronic incompatibility of the interfaces that leads to dangling bonds and trap states. These trap states in turn dominates the electronic behaviour and decouples the functional oxides from the underlying Si channel. For example, despite the pioneering work of epitaxial growth of a ferroelectric layer on silicon without a buffer layer in ref. 17, a direct and reversible control of the Si channel charge could not be achieved.

In the following, we present a fundamental advancement in the integration of such dissimilar materials. This is achieved by epitaxial transfer of single-crystalline functional oxides directly onto Si. Because of the fact that the process can be carried out at room temperature, it avoids the interface chemistry and thermal issues described above. We demonstrate transfer of functional oxides as thin as one unit cell (4 Å), which is almost three orders of magnitude thinner than any other transfer technique reported for complex oxides. The lattice structure, surface morphology, piezoelectric coefficient, dielectric constant, polarization switching and spontaneous and remnant polarization of the transferred ferroelectric oxide are commensurate with those of the as-grown films on lattice matched oxide substrates. Remarkably, when a transferred Pb(Zr_0.2_Ti_0.8_)O_3_ (PZT) is used as the gate of a silicon-on-insulator (SOI) transistor, it shows clear control of the channel charge with ferroelectric polarization evidenced in the signature anti-clockwise hysteresis loop and an abrupt jump in the current, attesting to high-quality interface and single-crystalline nature of the transferred film respectively. We also demonstrate transfer of single-crystalline superlattices and multiferroic heterostructures on Si that illustrate the tremendous flexibility offered by the technique reported in this work.

## Results

### Structural characteristics of complex oxides on silicon

**Figure 1:**
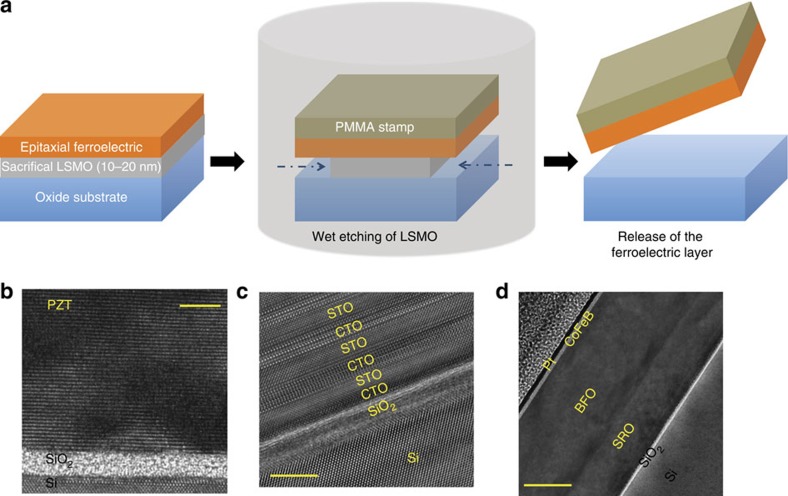
Epitaxial ferroelectric films on silicon. (**a**) Transfer process. Epitaxial thin films (one unit cell −100 nm) of ferroelectric oxides are grown on lattice-matched substrates with a thin (10–20 nm) sacrificial layer using pulsed laser deposition method. The stack is then immersed in a diluted KI+HCl solution, which isotropically etches La_0.7_Sr_0.3_MnO_3_. A polymethyl methacrylate handle is used to transfer the released ferroelectric layers onto Si and other substrates. Transmission electron microscopy images of the transferred (**b**) Pb(Zr_0.2_Ti_0.8_)O_3_, (**c**) (CaTiO_3_/SrTiO_3_)_6_ superlattices and (**d**) SrRuO_3_/BiFeO_3_/CoFeB/Pt multilayers on Si substrate. The scale bars are 5 nm in **b**,**c** and 40 nm in **d**.

**Figure 2:**
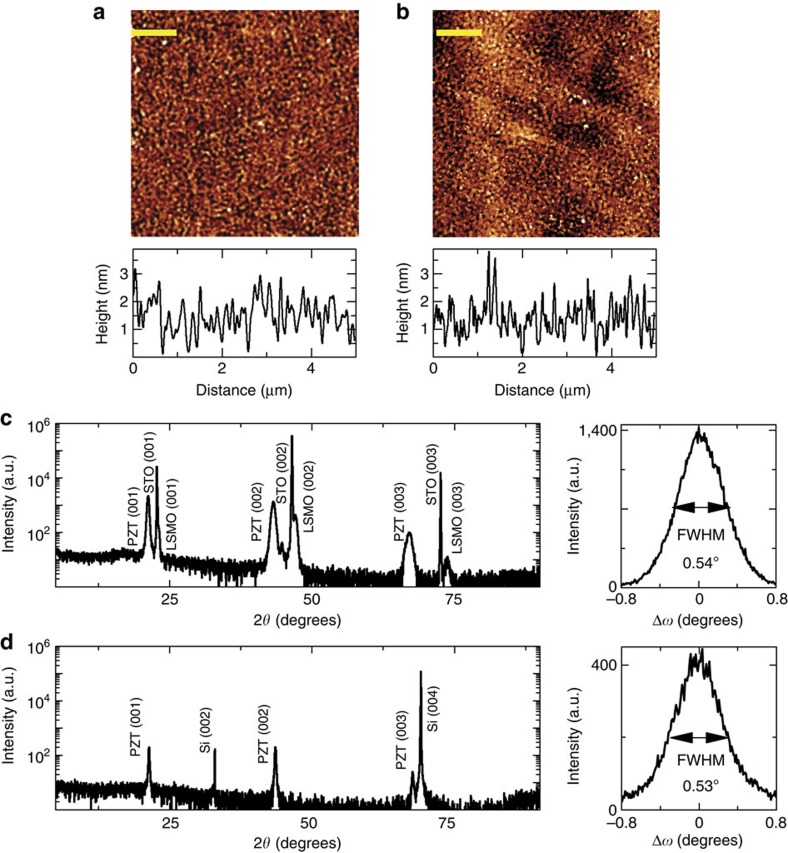
Structural characterization of the as-grown Pb(Zr_0.2_Ti_0.8_)O_3_ (PZT) and the transferred PZT on silicon. (**a**,**b**) Atomic force microscopy images of the top and bottom surfaces of transferred PZT. The top surface is probed when PZT is sitting on Si and the bottom surface is probed by placing PZT/PMMA bilayer inverted on Si. The RMS roughness of top and bottom surfaces is 0.61 and 0.67 nm, respectively. These are comparable to 0.41 nm roughness of the source PZT film's top surface (Supplementary Fig. 1). Scale bar, 1 μm. (**c**,**d**) *θ*-2*θ* scan and rocking curve around PZT (002) reflection peak of the source PZT on SrTiO_3_/ La_0.7_Sr_0.3_MnO_3_ substrate and transferred PZT on Si (001). The absence of any phase other than the 001 family of planes of Si and PZT points that the transferred PZT is single crystalline.

For epitaxial transfer, we start by growing single crystal, 0.4–100-nm thick PZT on 20 nm thick La_0.7_Sr_0.3_MnO_3_ (LSMO) coated SrTiO_3_ (STO) substrate by using pulsed laser deposition (PLD) (for structural properties see Supplementary Figs 1 and 2). Subsequently, the LSMO layer is wet etched. This releases the layer(s) sitting above it (Fig. 1a), which is then carried by a transfer stamp based on polymethyl methacrylate (PMMA) and placed on the target substrate such as Si. High-resolution transmission electron microscopy reveals atomically sharp interfaces and no interfacial layer when Si surface is properly passivated (Fig. 1b, Supplementary Fig. 3a,b). Similar results are obtained when stack with multiferroic (SrRuO_3_/BiFeO_3_/CoFeB/MgO) and superlattices (CaTiO_3_/SrTiO_3_)_6_ are transferred (Fig. 1c,d). Figure 2 shows the structural characteristics of transferred films of PZT on Si. The root mean square (RMS) roughness of the transferred PZT is 0.61 nm (Fig. 2a) which is comparable to that of the as-grown film (0.42 nm; Supplementary Fig. 1a). The bottom surface of the PZT, which was released from LSMO, shows a RMS roughness of 0.67 nm (Fig. 2b). This indicates that the surface morphology of PZT is insensitive to the etch chemistry and removal of LSMO. The *θ*-2*θ* scan of the transferred film using X-ray diffraction (Fig. 2d) is essentially identical to the as-grown film (Fig. 2c) and shows peaks only from the PZT (001) and Si (00 l) family planes, suggesting that the transferred PZT is a single crystal. The lattice constants for the as-grown and the transferred PZT are 4.14 and 4.15 Å respectively and the full width half maxima measured from the rocking curves are 0.54° and 0.53°. This suggests that the overall film quality remains intact after the transfer process. Similar behaviour is observed when PZT is transferred on other surfaces such as 5-nm amorphous Al_2_O_3_ coated Si, thermally grown amorphous SiO_2_ coated Si, sputter deposited amorphous Au coated Si, single-crystal oxide substrates such as LSMO on STO and so on (Supplementary Figs 3 and 4).

### Switching in single crystal Pb_0.2_Zr_0.8_TiO_3_ on silicon

**Figure 3:**
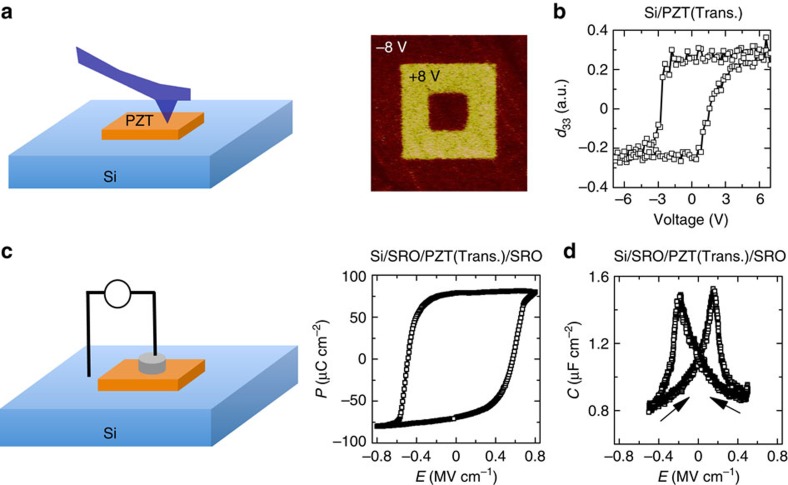
Piezoelectric and ferroelectric properties of the transferred PZT on Si. (**a**) Piezoforce microscopy of the transferred layer. The ferroelectric domains can be reversibly poled and the states are very stable. (**b**) The d_33_ coefficient of the transferred Pb(Zr_0.2_Ti_0.8_)O_3_ on Si. (**c**,**d**) *P*-*E* and *C*-*E* loop of a SrRuO_3_/ Pb(Zr_0.2_Ti_0.8_)O_3_/SrRuO_3_ transferred on highly doped Si substrate.

Next we studied the electromechanical properties of the transferred PZT using the piezoelectric force microscopy. As shown in Fig. 3a, the ferroelectric domains of the transferred PZT on Si could be reversibly poled by applying oppositely directed electric fields from the piezoelectric force microscopy tip. The domains, thus, obtained retained their respective polarization states even after 24 h. Figure 3b shows the *d*_33_-*V* loop for the transferred PZT on Si. The *d*_33_ amplitude is similar to that obtained in the as-grown film (Supplementary Fig. 5).

### Electronic transport properties of Pb_0.2_Zr_0.8_TiO_3_ on silicon

To understand the quality and applicability of the transferred PZT for electronic applications, we explore the polarization (*P*)-field (*E*) and capacitance (*C*)-*E* characteristics. Figure 3c,d shows the results for the case where an epitaxial tri-layer SrRuO_3_(SRO)/PZT/SRO heterostructure on LSMO buffered STO substrate was grown and subsequently transferred onto a Si substrate. The saturation polarization (∼75 μC cm^−2^) and the peak capacitance (∼1.6 μC cm^−2^) are similar to a typical as-grown film. The hysteresis is symmetric with the *V*=0 point because of a symmetric boundary condition on top and bottom for the PZT film^18^. Importantly, the results in Fig. 3c,d and Supplementary Fig. 6 demonstrate that the transfer method works equally well for multiple layers and therefore any arbitrary heterostructure can be transferred in this way. Monitoring the voltage across the ferroelectric after application of a pulsed voltage shows a transient decrease with time, characteristic of the intrinsic polarization switching^19^,^20^,^21^ (see Supplementary Fig. 7 for details).

### Single-crystal Pb_0.2_Zr_0.8_TiO_3_-gated Si transistor

**Figure 4:**
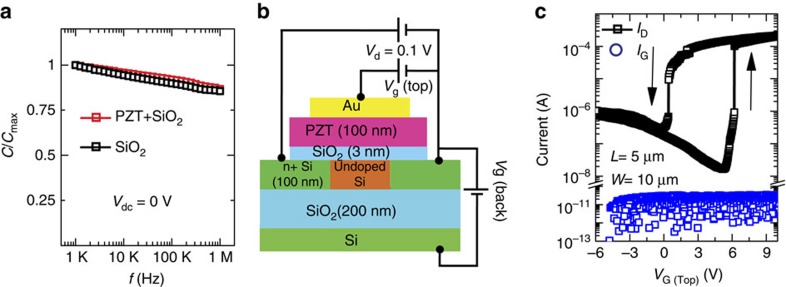
Single-crystal Pb(Zr_0.2_Ti_0.8_)O_3_ (PZT) gated silicon channel transistor. (**a**) Frequency-dependent capacitance of Si/SiO_2_ and Si/SiO_2_/transferred Pb(Zr_0.2_Ti_0.8_)O_3_. The capacitor size is 22 × 22 μm^2^. (**b**) Cross-sectional schematic diagram of the fabricated transistor on SOI substrate. The length, *L*, and width, *W*, of the silicon channel region are 5 and 10 μm, respectively, whereas gate electrode length is 20 μm. (**c**) *I*_D_−*V*_G_ (top gate) characteristics of the ferroelectric PZT-gated transistor at *V*_G_ (back gate)=0. The counter-clockwise hysteresis and two order of abrupt current change in the *I*_D_−*V*_G_ characteristics demonstrates the control of the channel charge by the polarization of the transferred PZT layer.

To check the electronic quality of the interface, we demonstrate a functional Si field-effect transistor with a transferred PZT layer as the gate oxide. We exploit one of the major strengths of the transfer process, namely, a single-crystalline ferroelectric can be transferred onto any arbitrary surface, such as Si/SiO_2_ (3 nm) surface. The Si/SiO_2_ interface ensures excellent surface for the channel and at the same time provides a large band-offset with the channel that stops hot electrons from easily tunnelling into the ferroelectric atop it. The PZT is then transferred onto the channel to form the gate. Figure 4a shows the normalized, frequency-dependent capacitance of a Si/SiO_2_ capacitor with and without the transferred PZT on top. The dispersion is identical for both, indicating that the transfer of PZT does not degrade the quality of the interface. The impedance angle is close to 90° for both capacitors over the entire frequency range. Similar behaviour is seen for Si/Al_2_O_3_ interfaces (Supplementary Figs 8 and 9). Figure 4b,c show the schematic representation of the fabricated transistor (optical image is shown in Supplementary Fig. 10) and the *I*_D_−*V*_G_ characteristics. There are two important points about the *I*_D_−*V*_G_ characteristic. Firstly, the *I*_D_−*V*_G_ shows counter-clockwise hysteresis for the n-type transistor which is a characteristic signature of the ferroelectric control of the charge. Secondly, the abrupt jump in the current indicates that the ferroelectric PZT switches abruptly as expected in a single-crystalline structure. The handedness of the hysteresis and the abruptness in the current together demonstrate the successful integration of a functional, single-crystalline oxide onto a Si device, a goal that has been long pursued but has so far been elusive^17^. All of the *I*_D_−*V*_G_ loops are repeatable (Supplementary Figs 11 and 12).

## Discussion

Our work is a fundamental advancement over prior transfer methods that have been explored before for ferroelectrics (such as the smart-cut techniques where only microns thick films have been transferred and a typical surface RMS roughness of 11–70 nm is observed^22^,^23^,^24^,^25^ due to ion damage. By contrast, we have integrated films with thickness much smaller than this roughness ranges down to a single unit cell. The generality of our approach paves the way to integrate complex oxides on not only Si but also other semiconductors such as GaN where the polarization of a single-crystalline ferroelectric could be used to counteract the built-in polarization. Epitaxially transferred semiconductors is a commercial technology^26^. This indicates that the reported technique should be scalable to commercially relevant sizes, thereby enabling many novel applications in electronics and multiferroic spintronics^26^,^27^,^28^,^29^,^30^,^31^.

## Methods

### SOI transistor with FE gate

We start with SOI wafer with a highly doped Si handle, a SiO_2_ box and a p-type Si with a thickness of∼100 nm as the active region. The Si handle is used as a back gate. First a mesa was defined and the source and drain regions were patterned giving a channel length of 5 μm and width of 10 μm. After that the source and drain regions were doped n^+^. Next, the Si mesa was covered by a 3 nm thick, thermally grown SiO_2_ layer. This provides excellent interface with the Si. Then a PZT flake was transferred onto the channel region. Finally the top gate was patterned (see also Supplementary Note 8).

## Additional information

**How to cite this article**: Bakaul, S. R. *et al*. Single crystal functional oxides on silicon. *Nat. Commun.* 7:10547 doi: 10.1038/ncomms10547 (2016).

## Supplementary Material

Supplementary Information Supplementary Figures 1-12, Supplementary Notes 1-8 and Supplementary References.
