# Supplementary material for: Single crystal functional oxides on silicon
Source: Nat Commun. 2016 Feb 8;7:10547. doi: 10.1038/ncomms10547 (PMC4748113; doi:10.1038/ncomms10547)
Supplement: Supplementary Information — Supplementary Figures 1-12, Supplementary Notes 1-8 and Supplementary References. [file ncomms10547-s1.pdf]

Supplementary Figures

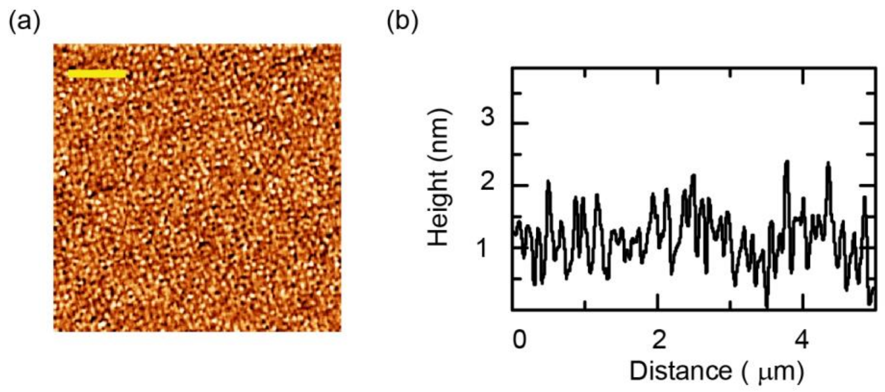

Supplementary Figure 1: Surface morphology of the source STO/LSMO20/PZT100 film. (a) Topography image. (**b)** Cross-sectional height profile. The scale bar is 1 μm.

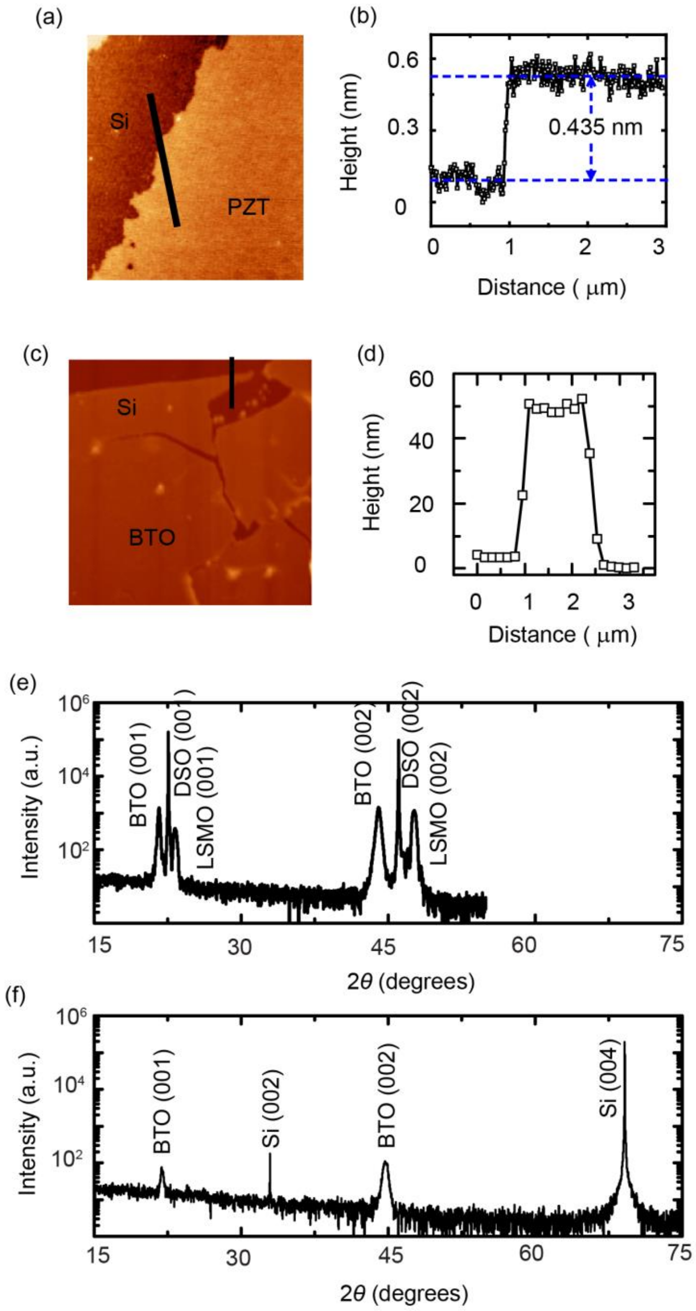

Supplementary Figure 2: Structural characterization of ultrathin PZT and BTO. (**a**) - (**b**) Surface morphology and cross-sectional height profile of the transferred 1 unit cell thick PZT on Si. (**c**) – (**d**) Surface morphology and cross-sectional height profile of the transferred 55 nm thick BTO on Si. The black line indicates the place where cross-sectional height profile is taken. (**e**) – (**f**) XRD θ-2θ scan of BTO film on DSO/LSMO substrate and transferred BTO on Si, showing the crystallinity of BTO remains intact after transfer process.

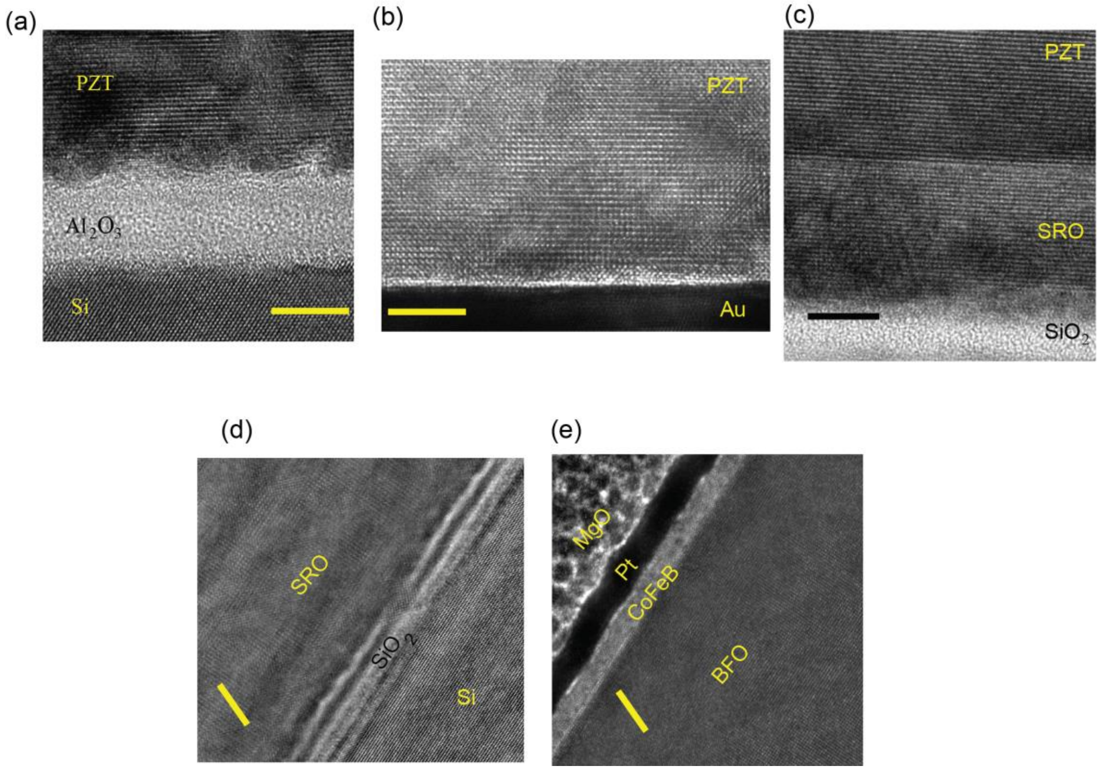

Supplementary Figure 3: TEM images. Transferred PZT on (**a**) Si/Al_2O_3 and (**b**) Si/Au surface. (**c**) Transferred SRO/PZT/SRO heterostructure on Si/SiO_2. (**d)** and (**e**) Bottom and top part of the transferred SRO/BFO/CoFeB/Pt on Si/SiO_2. The TEM images confirm that wide range of single crystal oxide materials can be pristinely transferred onto any type of substrate.

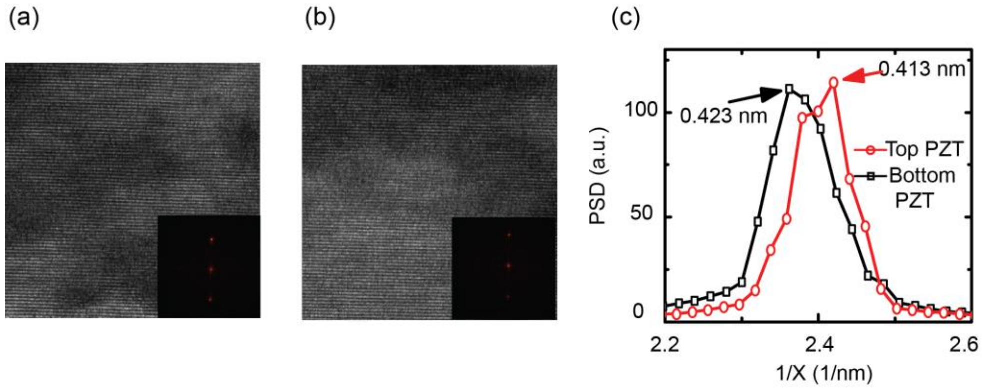

Supplementary Figure 4: Lattice constant extraction from the Fourier transformation (FT) of the TEM images of transferred PZT near top and bottom interface. (**a**)-(**b**) TEM images of transferred PZT's bottom and top portion, respectively. The size of the area analyzed is 32 nm × 32 nm. The insets show the color coded Fourier transformed images, where the distance of the brightest spot from the center is an inverse measure of the lattice constant. **(c)** Power spectral densities of the FT images, where the peak position corresponds to the most dominant frequency of the features present in the TEM images and its inverse is a direct measure of the average lattice constant. Clearly, the bottom portion of the transferred PZT is more compressively strained than the top portion.

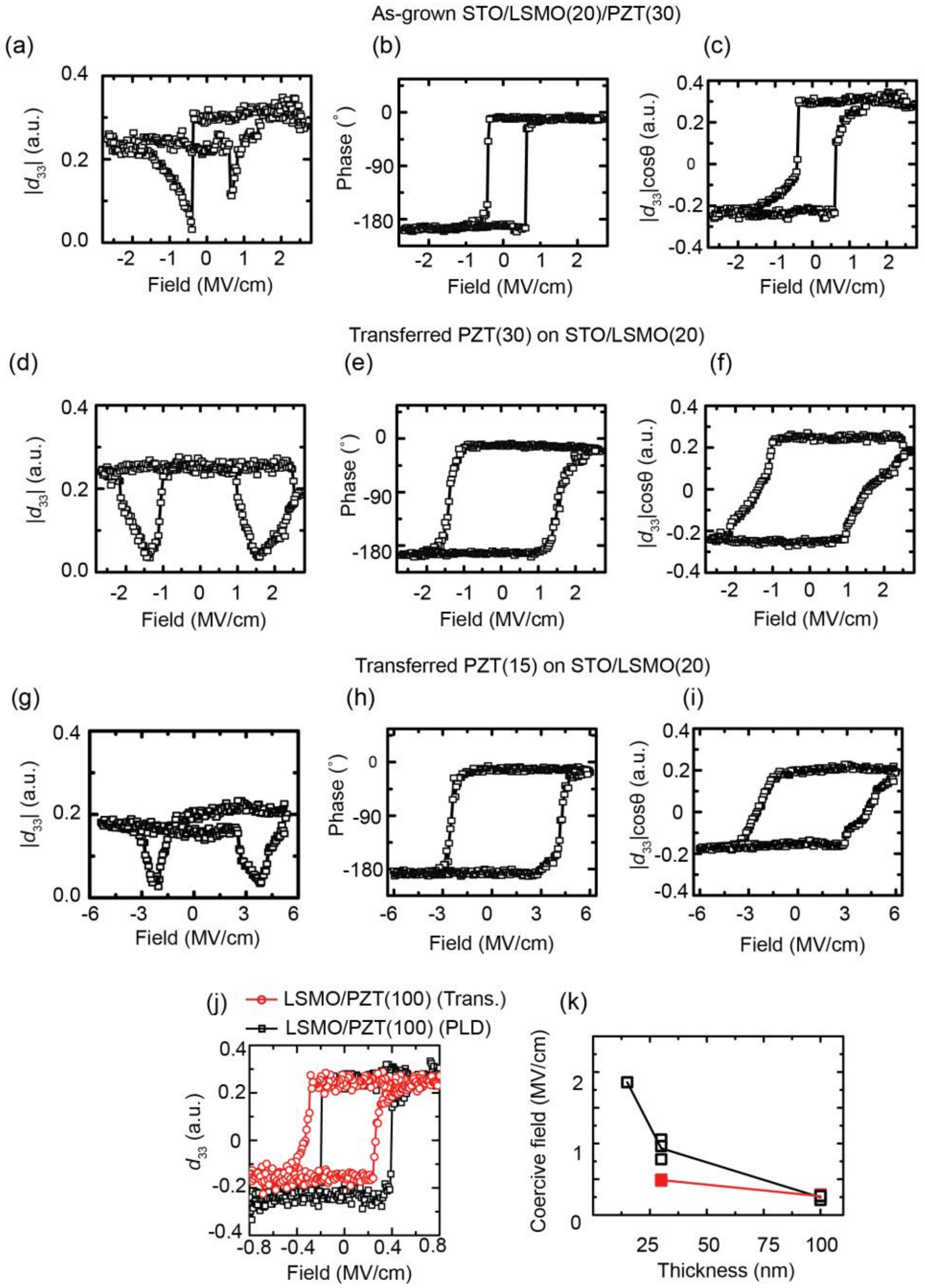

Supplementary Figure 5: Thickness dependence of the d_{33} coefficients of as-grown PZT and transferred PZT on same substrate. Amplitude, phase and $|d_{33}|\cos\theta$ of (**a**)–(**c**) as-grown STO/LSMO/PZT30, (**d**)-(**f**) transferred 30 nm thick PZT on STO/LSMO substrate and (**g**)-(**i**) transferred 15 nm thick PZT on STO/LSMO substrate. (**j**) Comparison between d_{33} coefficients of PLD-grown, 100 nm thick source PZT film on STO-LSMO substrate and transferred PZT on same substrate. d_{33} coefficients are similar in magnitude for both transferred and as-grown PZT, proving that transfer process does not significantly modify the electro-mechanical properties of PZT. (**k**) Thickness dependence of the coercive fields of as-grown and transferred PZT.

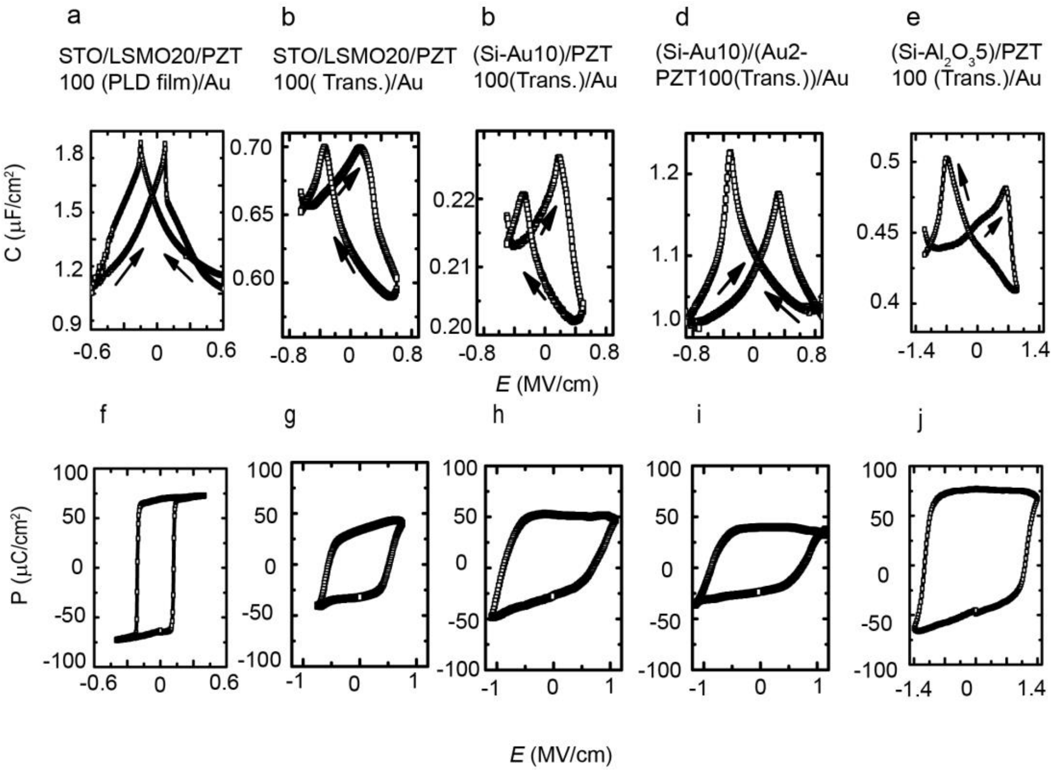

Supplementary Figure 6: *C-E* and *P-E* of as-grown and transferred PZT (100 nm thick) on different substrates. *C-E* loops of (**a**) the as-grown (by PLD) PZT film on STO/LSMO20, (**b**) the transferred PZT on STO/LSMO20, (**c**) the transferred PZT on Si/Au10, (**d**) the transferred PZT (with 2 nm evaporated Au at the bottom surface) on Si/Au10 and (**e**) the transferred PZT on Si/Al_2O_3 (5 nm), respectively. (**f**) – (**j**) *P-E* loops of the above mentioned samples.

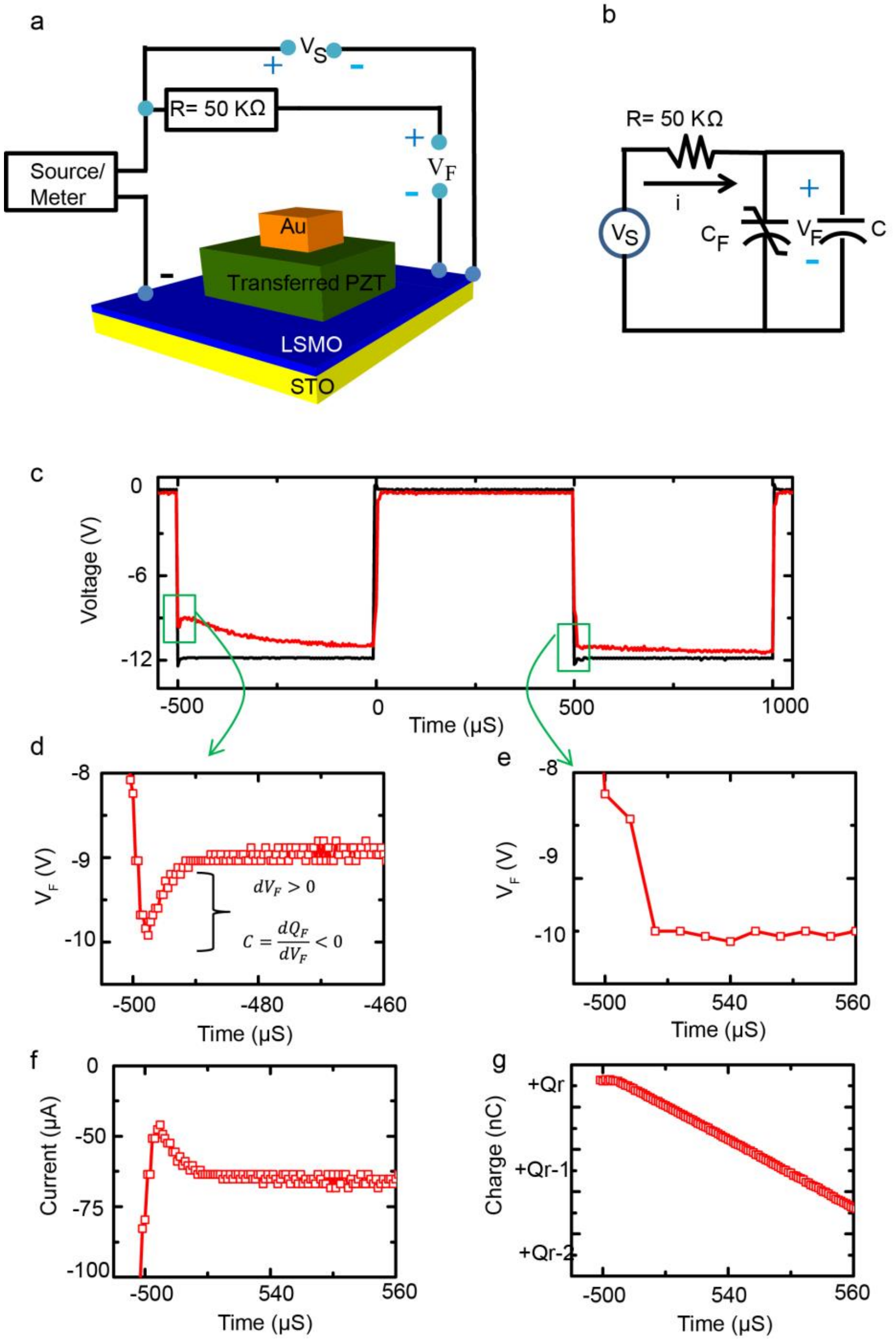

Supplementary Figure 7: Negative capacitance in transferred PZT on STO/LSMO substrate. (**a**) Schematic diagram of the transient characteristics measurement setup. (**b**) Equivalent circuit diagram. (**c**) Voltage waveform across the power source (V_S) and the FE capacitor (V_F). (**d**)–(**e**) Zoomed-in view of the transient response across FE during first and second pulse, respectively. (**f**) Charging current through FE. (**g**) Stored charge in FE.

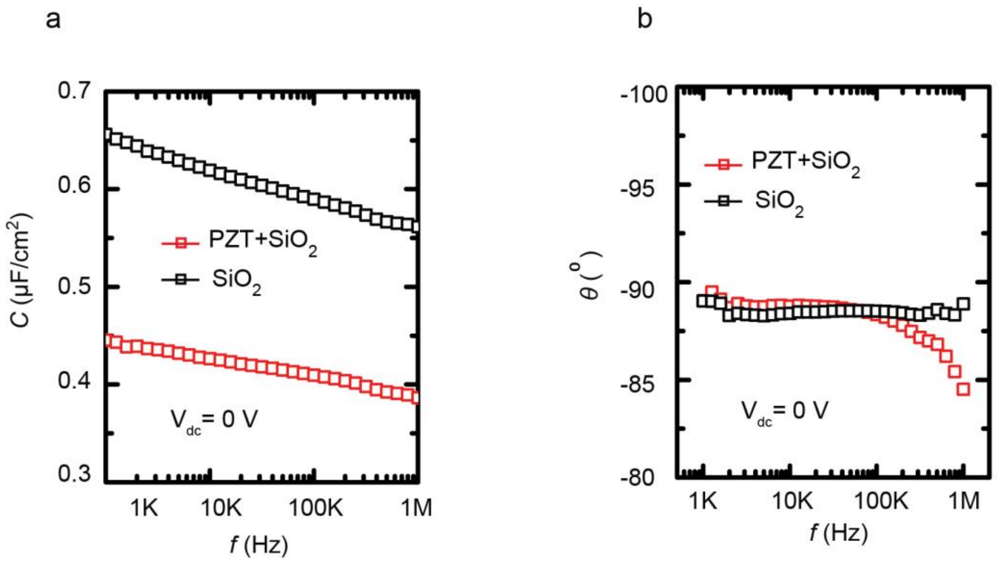

Supplementary Figure 8: *C*-*f* data. Frequency dependent (**a**) capacitance and (**b**) admittance angles of Si-SiO_2-transferred PZT and Si-SiO_2 capacitors.

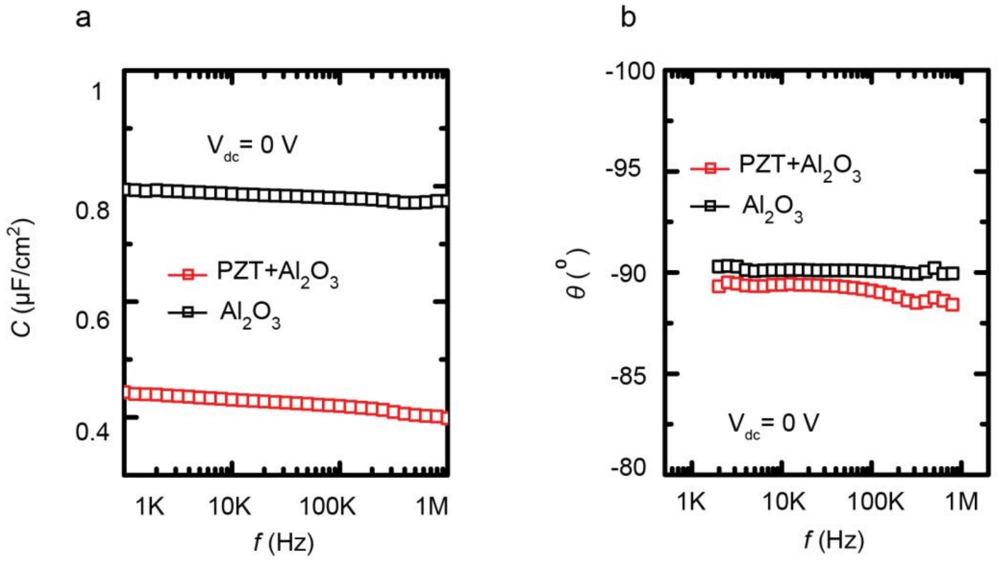

Supplementary Figure 9: *C*-*f* data. Frequency dependent (**a**) capacitance and (**b**) admittance angles of Si-Al_2O_3-transferred PZT and Si-Al_2O_3 samples.

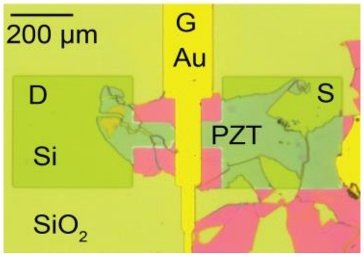

Supplementary Figure 10: SOI Transistor. Optical micrograph of the single crystal PZT gated Si transistor.

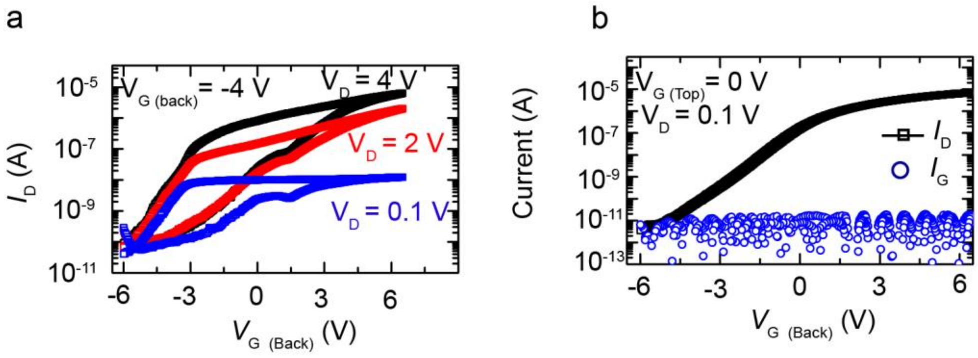

Supplementary Figure 11: I_D-V_G characteristics of FEFET. (**a**) I_D-V_G (top gate) characteristics of the ferroelectric PZT gated transistor at V_G (back gate)= -4 V for different V_D. (**b**), I_D-V_G (back gate) characteristics of the transistor.

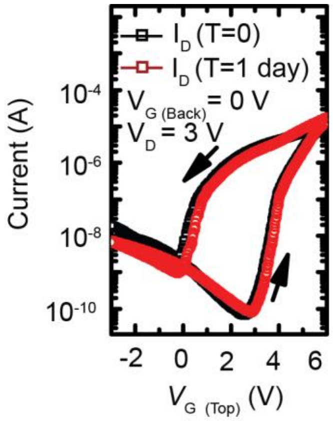

Supplementary Figure 12: Retention. I_D-V_G (top gate) characteristics of a FEFET (channel length and width 5 μm) measured at a time gap of 1 day.

Supplementary Note 1: Materials growth by pulsed laser deposition (PLD)

STO/LSMO20/PZT: 20 nm of LSMO is grown on the STO (001) substrate at 750 °C with repetition rate of 2 Hz. The film is cooled down to 630°C at a rate of 5°C /min. PZT is grown at this temperature using 10 HZ repetition rate. Both the layers are grown at the oxygen background pressure of 100 mTorr and laser energy density of 1 J/cm^2. The film is cooled down to room temperature at the rate of 5°C /min in 1 atm pressure of oxygen. Supplementary Figure 1a and Supplementary Figure 1b show the surface morphology of PLD grown STO/LSMO20/PZT100 sample, which is used as the source substrate for the transferred PZT flakes shown in main text Fig. 2.

STO/LSMO20/SRO15/PZT60/SRO15: After growing 20 nm of LSMO at 750 °C on the STO (001) substrate, the film is cooled down to 700 °C (cooling rate 5 °C/min) where 15 nm of SRO is grown at 10 HZ. Then the film is cooled down to 630 °C where 60 nm of PZT is grown. After the PZT growth 15 nm of SRO is grown at same temperature. All the layers are grown at the oxygen background pressure of 100 mTorr and laser energy density of 1 J/cm^2. The film is cooled down to room temperature at the rate of 5 °C/min in 1 atm pressure of oxygen.

DSO/LSMO20/BTO: 20 nm LSMO is grown following the same recipe as described earlier. Then the film is cooled down to 600 °C at a rate of 10 °C/min and BTO is grown at that temperature. The oxygen background pressure is 20 mTorr, laser energy density is 1.5 J/cm^2 and repetition rate is 10 Hz. The film is cooled down to room temperature at the rate of 10 °C /min in 1 atm pressure of oxygen.

Superlattices: Superlattices of $SrTiO_3$/$CaTiO_3$ were synthesized using Reflection High Energy Electron Diffraction (RHEED) - assisted PLD. To ensure stoichiometric transfer of STO and CTO, growth temperature was set at 700 C and growth pressure was 50 mTorr with both targets being ablated by a laser fluence of 1.5 J/cm^2. The growth was monitored using RHEED with Frank-van der Merwe layer-by-layer growth mode present throughout the process.

$DyScO_3$/$SrRuO_3$40/$BiFeO_3$70/CoFeB4/Pt4: These films were prepared on single-crystalline (110) $DyScO_3$ substrates by PLD. For SRO and BFO films substrate temperatures were 690 °C and 700 °C and Oxygen pressures were 50 mTorr and 100 mTor , respectively. The films were grown at a repetition rate of

8 Hz with a laser fluence of 1.1 $J.cm^{-2}$. After growth, the samples were cooled to room temperature in an Oxygen pressure of 750 Torr.

Supplementary Note 2: Details of single crystal FE transfer process

The key to transfer single crystal FE onto Si is using a suitable sacrificial layer which, a) allows the crystalline growth of the FE layer on top of it and b) can be selectively etched away without affecting the ferroelectric layer. Fig. 1 shows the steps involved in the transfer process. We grow single crystal PZT on 20 nm thick LSMO coated STO substrate by using PLD. Then poly methyl methacrylate (PMMA) layer is spin coated, which serves as the transfer stamp. We chose PMMA over other typical stamps such as PDMS, since the adhesion between PZT and PMMA is better than that between PZT and PDMS. Moreover, PMMA can be easily and cleanly removed by organic chemical such as acetone which helps to get better yield in transferring nano materials (*1*). PMMA 950A4 is spin coated at 4000 rpm for 30 seconds, followed by a baking at 120 °C for 1 minute, which leaves a uniform film of 200-250 nm thickness. The second and the most important task to successfully transfer single crystal PZT is finding an etchant which removes only the sacrificial layer LSMO and does not react with the PMMA stamp and PZT. We use KI (4 mg) + HCl (5 mL) + H_2O (200 mL) for etching (*2*). Since LSMO/PZT stack is covered by PMMA stamp, LSMO is slowly etched away only from the side. The possible chemical reaction between LSMO and the etchant is:

$$La_{0.7}Sr_{0.3}MnO_3+H_2O+5\ HCl+\ KI = MnCl_2 + Cl_2 + \tfrac{1}{2}\ I_2 + KOH + 3\ H_2O + La_{0.7}Sr_{0.3}Cl$$

After 12 hours, PZT/PMMA stack is released and collected from the solution. Then we wash it by DI water and dry up by N_2 gas. The stamp is transferred by using micromanipulator and a gentle force on the target substrate, followed by removal of PMMA by acetone. Following this procedure we have transferred various types of ferroelectric materials and heterostructures such as PZT, BTO, CTO/STO superlattices, SRO/$BiFeO_3$/CoFeB/Pt and SRO/PZT/SRO. The transfer process works equally well for all of these structures, including 1 unit cell thick PZT. Supplementary Figure 2a and Supplementary Figure 2b show the topography images of the transferred 1 unit cell thick PZT on Si. The height profile clearly shows a step of approximately 0.435 nm between Si and PZT flake, where the error bar is ±0.1 nm. This step height

matches with the estimated thickness found from the growth rate of the source film, confirming the unit cell transfer capability of our technique. Supplementary Figure 2c and Supplementary Figure 2d show topography images of the transferred BTO on Si. The XRD ө-2ө scan shown in Supplementary Figure 2e and Supplementary Figure 2f clearly demonstrate the single phase of BTO before and after transfer process.

Supplementary Note 3: TEM images of transferred oxide layers

Supplementary Figure 3a-c show the TEM images of the transferred PZT on 5nm Al_2O_3 coated Si, 10 nm Au coated Si and transferred SRO/PZT/SRO heterostructures on Si/SiO_2, respectively. The images reveal atomically sharp interfaces. The SiO_2 layer is necessary for transistor application and could be easily avoided by performing the transfer in an inert environment. The area near top surface of PZT also reveals no microstructural damage, as shown in Supplementary Figure 4b. All these TEM images points that wide range of materials including multilayers can be placed on arbitrary substrate in pristine condition.

Supplementary Note 4: Extracting lattice constant of the bottom and top portion of transferred PZT on Si/SiO_2 by Fourier Transformation of the TEM image

Supplementary Figure 4a-b show the TEM images of 32×32 nm area of transferred PZT near the bottom and top surface, respectively. The spacing between the periodic lines seen in the TEM image, stemming from the repetitive arrangement of PZT planes is a direct measure of the c-axis lattice parameter of PZT. To determine the exact periodicity from the images we perform Fourier transformation on them and the resultant color coded images are shown in the insets of Supplementary Figure 4 and Supplementary Figure 4b. The two brightest correspond to the periodicity of the planes in reciprocal space. To get a quantitative value of the lattice periodicity we calculate the power spectral densities (PSD) by WSxM (*3*), which basically performs squared fast Fourier transformation (*4*) of the grey scale surface image:

$$PSD\ (f_x, f_y) = \lim_{d\to\infty} \frac{1}{d^2} \left| \int_{-d/2}^{d/2} \int_{-d/2}^{d/2} S(x,y) \times \exp\left[-2\pi i\left(x f_x + y f_y\right)\right] dxdy \right|^2$$

Here $S(x, y)$ represents the 2 dimensional matrix of the color values of the TEM image, f_x and f_y are the spatial frequencies in x and y direction, respectively. The WSxM software also directly provides 1 dimensional PSD for digitized data in the vector direction where the intensities are highest, which is shown in Supplementary Figure 4c. The inverse of the peak position in this PSD spectra is the lattice constant of PZT, which is 4.23 Å for the bottom portion and 4.13 Å for the top portion. The difference between lattice constants near two surfaces indicates that during growth the PZT near LSMO was more compressively strained than PZT near top surface.

Supplementary Note 5: d_{33} Coefficient of PZT

All the d_{33} coefficient measurements are done in a local configuration, i.e. without any global top electrode. The scanning probe microscopy tip is used to apply the dc+ac electric field between PZT and the grounded substrate (Si or LSMO). Supplementary Figure 5a-c show the $|d_{33}|$, phase and $|d_{33}|\cos\theta$ loops of PLD grown, 30 nm thick PZT film on 20 nm LSMO coated STO substrate. For a comparison, PZT from this source substrate is transferred onto another similar STO/LSMO20 substrate and the d_{33} loops are measured by exactly same tip as that is used to measure the source film. Supplementary Figure 5d-f show the d_{33} loops of the transferred 30 nm thick PZT. An interesting point to note is that the coercive field is significantly increased from 0.5 MV/cm to 1.1 MV/cm after transferring. We have also successfully transferred very thin (15 nm) PZT on LSMO substrate. The d_{33} loops of the transferred thin PZT are shown in Supplementary Figure 5g-i. The coercive field of the transferred 15 nm thick flake is 1.85 MV/cm. The enhancement of coercive field is not observed for the thick PZT (100 nm) samples, as shown in Supplementary Figure 5j. However, all of the d_{33}-V loops are found to be asymmetric with respect to V=0 axis, which is presumably due to the work function difference between the dissimilar contact materials. A comparison between the coercive fields extracted from d_{33} measurements of the as-grown and transferred PZT samples are shown in Supplementary Figure 5k.

Supplementary Note 6: Capacitance and polarization characteristics of transferred PZT on different substrates

To measure the *C*-*E* and *P*-*E* characteristics, 20×20 μm^2 sized Au pads are fabricated on top of the PZT flakes by using shadow mask and e-beam evaporator. The positive terminal is put on top of the Au pad and the conducting substrate (highly doped Si or LSMO) serves as the ground terminal. The Si substrate is p+ doped with doping concentration of 2×10^{19}/cm^3. Agilent B1500 and Radiant ferroelectric tester are used for *C*-*E* and *P*-*E* measurements, respectively. For capacitance measurement, an ac signal with 200 mV p-p amplitude and 100 KHz frequency is superimposed with the dc bias. The high frequency reduces the effect of the defects and trap charges. Supplementary Figure 6a-c show the *C*-*E* of the as-grown film grown on STO/LSMO20, transferred PZT on STO/LSMO20 and Si/Au10 substrate, respectively. All the figures are asymmetric with respect to *E*=0 axis, which is presumably due to the asymmetric electrodes, although the transferred PZT on Si/Au10 is expected to have symmetric boundary conditions and show symmetric *C*-*E*. This happens because the top contact Au is directly evaporated on PZT, but the bottom contact is not, resulting in different boundary conditions. When both top and bottom contacts are made by directly evaporating Au on PZT, the asymmetry disappears, which is shown in Supplementary Figure 6d. In this case, before transferring on Si/Au, 2 nm Au is evaporated at the back side of PZT (while PZT is on the stamp). Supplementary Figure 6e shows the *C*-*E* of the transferred PZT on 5 nm Al_2O_3 coated Si. The *P*-*E* loops of these samples are shown in Supplementary Figure 6f-j. The saturation polarization value remains similar. For PZT transferred on Si/Al_2O_3 substrate both *C*-*E* and *P*-*E* loops become elongated in the horizontal (voltage) axis. This is due to the additional voltage drop that happens across the Al_2O_3 layer. The capacitance on the Si/Al_2O_3 is also smaller due to the same reason. Nonetheless, both *P*-*E* and *C*-*E* show excellent ferroelectric behavior.

Supplementary Note 7: Transient electronic response of the transferred PZT: observation of negative differential capacitance

One of the most attractive properties of the FE material is the negative differential capacitance which has huge potential for energy harvesting applications and reducing the sub-threshold swing in field effect transistors (*5*,*6*). Capacitance is defined as $C = \frac{dQ}{dV} = [{d^2U}/{dQ^2}]^{-1}$, where V, U and Q are the voltage, energy and charge, respectively. The negative capacitance can be understood from the curvature of the double well shape of the energy landscape originated from the Landau's model of ferroelectric materials (*7*). Although predicted several decades ago, direct observation of negative capacitance has been reported very recently (*8*). By following the experimental techniques stated in Ref. 8 we have been able to observe the similar phenomenon in the transferred PZT on STO/LSMO20 substrate.

Supplementary Figure 7a and Supplementary Figure 7b show the negative capacitance measurement setup and the equivalent circuit, respectively. A 50 KΩ series resistance is added to the FE capacitor and the voltages across the source Agilent 81150a (V_S) and FE (V_F) have been probed by digital oscilloscope with 100X probes. The parasitic capacitance from the measurement setup is 40 pF. The system is initialized by a +12 V pulse. Then we apply two consecutive negative pulses with 500 µs pulse width: 0 V→-12 V→0 V→-12 V→0V. During the first negative pulse the FE polarization switches and while switching, it traverses through negative capacitance branch. This is manifested by the positive slope of the V_F-t curve in Supplementary Figure 7d during the time period from -498 µs to -491 µs. In this time the accumulated charge-time curve has a negative slope, which results in $C = \frac{dQ}{dV} < 0$. Since the FE does not switch during the second negative pulse, we do not observe the negative capacitance and positive slope branch in V_F-t.

Supplementary Note 8: Single crystal ferroelectric gated field effect transistor on Si

Interface: For field effect transistor it is imperative that the interface with the gate oxide is as much defect-free as possible. Measurement of the frequency (f) dependent capacitance of the gate material-Si

stack is a vital tool to probe the defect states. At low frequency the interface trap states contribute to the total capacitance whereas at high frequency excitation their effect is negligible. We transferred PZT (100 nm) on to conductive Si, which was covered by 6 nm of thermally grown SiO_2. The thickness of the SiO_2 layer is confirmed by the TEM image. Supplementary Figure 8a shows the *C*-*f* of the Si-SiO_2 only and Si-SiO_2-PZT stack, measured at zero dc bias and by 200 mV p-p ac voltage. The slopes of the *C*-*f* curves are very similar, indicating that the transfer of PZT does not alter the defect states at Si/SiO_2 interface. It also suggests that PZT does not bring in extra trapped charge to the system. The admittance angles, derived from the conductance and capacitance are close to 90° for both cases. Similar behavior has been observed for the transferred PZT on Si/Al_2O_3 (5 nm) and Al_2O_3 capacitors on Si (Supplementary Figure 9a and Supplementary Figure 9b).

Device fabrication: N-channel fully depleted silicon on insulator (FD-SOI) MOSFETs were fabricated on lightly doped p-type (~10^{16}/cm^3) silicon-on-insulator (SOI) wafers with buried oxide (BOX) thickness of ~200nm. Thermal oxidation is used to thin the SOI layer down to ~100nm. The active area is patterned by optical lithography followed by dry etching. A sacrificial thermal oxide (~3nm) is grown to reduce the etch damage. After using diluted HF to remove the sacrificial oxide, a 3nm gate thermal oxide was grown immediately. The channel region is patterned by optical lithography, and then ion implantation is performed to dope the source/drain regions n-type (5×10^{15} As^+/cm^2 at 80keV, 7° tilt). Rapid thermal annealing (20s @ 900 °C in N_2) is used to activate the dopants. Forming gas annealing (25 minutes @ 350 °C) was performed to improve Si/SiO_2 interface properties. Then PZT with the stamp is transferred on the channel region. The PMMA is washed away by acetone, leaving PZT on the channel region. Subsequently gate electrode patterning by optical lithography and Au deposition by thermal evaporation were done.

Supplementary Figure 10 shows the optical image of a typical transistor. We fabricated a number of transistors and in this Supplementary section we show the results of a transistor different than the main text's one. Supplementary Figure 11a shows the I_D-V_G (top gate) at V_G (back gate) =-4V at different V_D. Note that the ON current increases with increasing V_D. All the curves show anti-clock wise hysteresis. The shape of the loop can be understood by noting the following: When the transistor is ON, the ferroelectric is

essentially between two metal plates. Therefore, the switching of the polarization happens just it does in a usual capacitor structure and a reasonably sharp transition is seen when the transistor channel turns OFF. On the other hand, starting from OFF, the ferroelectric is between a metal plate (top electrode) and an insulator. The insulator slowly turns into a metal as the voltage across it is increased. Therefore, the actual voltage drop across the ferroelectric varies non-linearly as the total gate voltage is increased. Hence the transition from OFF to ON stretches out and shows a slower transition from OFF to ON than from ON to OFF. When top gate is grounded and only back gate voltage is swept, no hysteresis is seen in the I_D-V_G (see Supplementary Figure 11b) indicating that the observed hysteresis for the top gate sweep indeed comes from the ferroelectric polarization.

Supplementary Figure 12 shows I_D-V_G (top gate) characteristics of a different sample where two different I_D-V_G sweeps were performed with a time gap of 1 day. The two measurements show identical behavior, indicating that the polarization retention is robust and its effect on the Si channel is repeatable.

Supplementary References

1. L. Jiao *et al*., Creation of nanostructures with Poly (methyl methacrylate)-mediated nanotransfer printing.*J. Am. Chem. Soc*. **130**, 12612 (2008).

2. G. Bridoux, *et al*., An alternative route towards micro- and nano-patterning of oxide films. *Nanotechnology* **23**, 085302 (2012).

3. I. Horcas *et al*., WSXM: A software for scanning probe microscopy and a tool for nanotechnology. *Rev. Sci. Instrum*.**78**, 013705 (2007).

4. A. Duparré *et al*., Surface characterization techniques for determining the root-mean-square roughness and power spectral densities of optical components. *Applied Optics* **41**, 154-171 (2002).

5. S. Salahuddin, S. Datta, Use of negative capacitance to provide voltage amplification for low power nanoscale devices. *Nano Lett*. **8**, 405-410 (2008).

6. V. V. Zhirnov, R. K. Cavin, Negative capacitance to the rescue? *Nat. Nanotech*. **3**, 77–78 (2008).

7. M. E. Lines, A. M. Glass, *Principles and Applications of Ferroelectrics and Related Materials* (Clarendon, 2001)

8. A. I. Khan *et al.*, Negative capacitance in a ferroelectric capacitor. *Nat. Mat.* **14**, 182-185 (2015).
